# Gastrointestinal Complications in 147 Consecutive Patients with Peritoneal Surface Malignancy Treated by Cytoreductive Surgery and Perioperative Intraperitoneal Chemotherapy

**DOI:** 10.1155/2011/468698

**Published:** 2011-10-16

**Authors:** Angela Casado-Adam, Robert Alderman, O. Anthony Stuart, David Chang, Paul H. Sugarbaker

**Affiliations:** ^1^Washington Cancer Institute, Washington Hospital Center, Washington, DC 20010, USA; ^2^General Surgery and Gastroenterology Department, Reina Sofia University Hospital, Avenida Menéndez Pidal s/n., 14004 Córdoba, Spain; ^3^Westat, Rockville, MD 20850-3129, USA

## Abstract

Cytoreductive surgery (CRS) combined with hyperthermic intraperitoneal chemotherapy (HIPEC) is increasingly used in the treatment of peritoneal carcinomatosis from gastrointestinal malignancies. The purpose of this study is to reevaluate the incidence of gastrointestinal events and identify risk factors associated with this treatment approach. Between January 1, 2006 and December 31, 2009, 147 patients with appendiceal and colorectal carcinomatosis were treated. Gastrointestinal events were analyzed. The overall incidence of grade I–IV gastrointestinal events was 17%. There were 4 grade III gastrointestinal events that occurred in 4 patients and 11 grade IV gastrointestinal events that occurred in 8 patients. On univariate analysis of grade I–IV events a statistically significant association was observed with the following variables: histological grade, peritoneal cancer index (PCI), small bowel resection, colorectal anastomosis, and the number of anastomoses performed per patient. By multivariate analysis, PCI was identified as the only independent risk factor for gastrointestinal complications. CRS combined with a uniform HIPEC regimen is associated with a 17% gastrointestinal morbidity rate (grade I–IV). The frequency of gastrointestinal complications was associated with a large extent of disease measured by PCI (>30).

## 1. Introduction

In the past, peritoneal carcinomatosis (PC) was considered as a final stage of unresectable cancer with a short duration of survival [1–3]. Since the mid 1990s, studies on CRS combined with perioperative intraperitoneal chemotherapy (PIC), hyperthermic intraperitoneal chemotherapy (HIPEC), and/or early postoperative intraperitoneal chemotherapy (EPIC) are considered a new treatment options for selected patients with PC and peritoneal mesothelioma [4–12].

As the surgical technology has improved and the regimens for administering chemotherapy have become safer, the complications associated with this treatment approach have decreased [13–18]. In a systematic review, Chua reviewed all the relevant studies reported before August 2008 and concluded that the morbidity and mortality of CRS and HIPEC were similar to other major gastrointestinal interventions [19]. An important concept, “the learning curve,” has been demonstrated to operate in the expanded application of CRS and HIPEC [20–22]. Smeenk et al. reported the results of 323 procedures over a 10-year time period; they showed a decrease in major morbidity from 71% to 34% [22]. Yan et al. demonstrated a reduction in the rate of severe morbidity, transfusion requirement, duration of operation, and length of intensive care unit stay over a similar period of time in 140 patients [21].

In recent publications overall grade III-IV morbidity rates are shown to be between 7 and 41% [23–26]. For gastrointestinal events, small bowel perforations and anastomotic leaks are the most common and clinically significant complications after CRS and PIC [23–27]. The aim of this study was to report the incidence of gastrointestinal events and identify the associated risk factors.

## 2. Materials and Methods

### 2.1. Patient Characteristics

All patients with appendiceal and colorectal carcinomatosis treated in a uniform manner at Washington Hospital Center, between January 1, 2006 and December 31, 2009 constituted the basis of the present study. Institutional Review Board approval was obtained to collect and analyze these data. All patients with appendiceal and colorectal malignancy who received CRS combined with a standardized treatment with PIC were included (Table 1). Patients who had an incomplete CRS combined with PIC were included in the study, patients who had an open and close procedure or palliative debulking were not included. The quantitative prognostic indicators were the histological grade, the prior surgical score (PSS), the peritoneal cancer index (PCI), and the completeness of cytoreduction score (CC) [28].

### 2.2. Cytoreductive Surgery and Perioperative Intraperitoneal and Systemic Chemotherapy

The goal of surgery in these patients was to visibly clear the abdomen and pelvis of cancer nodules. This required a series of peritonectomy procedures and visceral resections (Table 1). Normal peritoneum or normal visceral structures were not resected. A mechanical bowel preparation was used in all patients. Within one hour prior to the abdominal incision, patients received antibiotic prophylaxis. Prophylaxis for venous thrombosis and pulmonary embolus during the cytoreductive surgery was limited to sequential compression devices (SCD Response, Kendall Co., Mansfield, MA).

All patients received HIPEC in the operating room immediately after the CRS but before intestinal anastomoses or repair of seromuscular tears. A combination of two drugs was administered intraperitoneally: mitomycin C (15 mg/m^2^) and doxorubicin (15 mg/m^2^). The target temperature for the entire abdomen during HIPEC was 41.5°C. Simultaneously, intravenous 5-fluorouracil (400 mg/m^2^) and leucovorin (20 mg/m^2^) were administered as a rapid infusion over 6–8 minutes. HIPEC was given using the Coliseum technique [29]. A heater circulator (Belmont Instruments Corporation, Billerica, MA) was used to maintain moderate hyperthermia within the abdomen and pelvis (41–43°C). During the first 45 minutes, only manual distribution of the chemotherapy solution occurred. In the second 45 minutes of the 90-minute treatment, seromuscular tears were repaired, the anterior and posterior rectus sheath were brought together with a running 2-0 Vicryl suture (Ethicon, Cincinnati, OH), and chest tubes were positioned.

Esophago-jejunal and colorectal anastomoses were performed with a 28 mm or 33 mm diameter circular stapler, respectively (Ethicon, Cincinnati, OH). Esophago-jejunal anastomoses were reinforced with a layer of silk sutures. If the colorectal stapled anastomoses could be well visualized, a second layer of 3–0 silk sutures was used to plicate the anastomosis. If this double layer anastomosis was performed, no diverting ostomy was constructed. If the colorectal anastomosis was too low to place a second layer of silk sutures over the circular stapled anastomosis, a diverting ostomy was constructed. All small bowel, colo-colic and ileocolic anastomoses were double-layer hand sewn with an inner layer of running 3-0 Maxon (Davis and Geck, Danbury, CT) and interrupted outer layer of 3-0 silk.

### 2.3. Early Postoperative Intraperitoneal Chemotherapy (EPIC)

The EPIC 5-fluorouracil (5-FU) was withheld in patients, who had a full course of oxaliplatin-based FOLFOX chemotherapy prior to CRS. The dose of EPIC 5-FU was 400 mg/m^2^/day for women and 600 mg/m^2^/day for men. It was infused via a Tenckhoff catheter over approximately 15 minutes with a 23-hour dwell time. After one hour of drainage, another administration of 5-FU occurred for 4 days after surgery

### 2.4. Postoperative Management

Patients were transferred directly to a surgical intensive care unit for monitoring and extubation. An 18 French nasogastric tube (Silicone Salem Sump Tube, Kendall Co., Mansfield, MA) was placed intraoperatively in all patients and remained until the drainage of bile from the stomach has ceased and some enteric function per rectum or per ostomy had occurred. All patients received postoperative intravenous feeding through the intrajugular vein for the five postoperative days and then through a percutaneous central catheter (Vaxcel, Glen Falls, NY) until gastrointestinal function returned. Closed suction drains remained in place after surgery in all patients until drainage was below 50 mL per 24 hours from a single drain.

### 2.5. Database for Morbidity Assessment

The database was specially constructed to evaluate gastrointestinal complications in patients with peritoneal surface malignancy. The morbidity variables were prospectively recorded according to the Common Toxicity Criteria (version 3.0) of the National Cancer Institute [30]. It consisted of 11 gastrointestinal adverse events (anastomotic failure, fistula, pancreatic fistula, pancreatitis, bile leak, chyle leak, prolonged ileus, small bowel obstruction, nausea/vomiting, diarrhea and ascites). Types of gastrointestinal adverse events observed and the grade are listed in Table 2. 

The following clinical variables were analyzed to asses factors predictive of gastrointestinal complications: gender, age (≤50 versus >51), primary cancer location, grade (grade 1 versus grade 2-3), prior surgical score (0–2 versus 3–5), peritoneal cancer index (0–10, 11–20, 21–30, 31–39, and 0-20 versus 31–39), completeness of cytoreduction (complete versus incomplete), peritonectomy procedures (pelvic, right upper quadrant, left upper quadrant, omental bursa, and anterior abdominal wall), number of peritonectomy procedures per patient (0–2 versus 3–5), visceral resections performed (omentectomy, splenectomy, rectosigmoid colon resection, right colon resection, hysterectomy, small bowel resection, transverse colon resection, and gastrectomy), visceral resections performed per patient (0–2 versus 3–7), types of anastomoses performed (esophago-jejunal, small bowel, ileocolic, colocolic, and colorectal), number of anastomoses performed per patient (0–2 versus 3–5), ostomies performed (none, diverting ileostomy and end ileostomy), blood replacement (none, 1–3 units, 4–6 units, >6 units), fresh frozen plasma replacement (none, 1–4 units, >4 units), time in the operating room in hours (0–6, 7–12, >12), and chemotherapy treatment (HIPEC only versus HIPEC plus EPIC).

### 2.6. Statistical Methods

For univariate methods to assess the association between gastrointestinal complications and pre- and perioperative clinical characteristics, the Pearson Chi-square was used, or the Fisher's exact test was used if there was sparse distribution. Those clinical characteristics that were significantly correlated to the outcome by univariate analysis (*P*-value < 0.05) were then fitted into the logistic regression model for multivariate analysis of variances to assess the strength of the risk factors.

All statistical analyses were conducted using SAS (SAS Institute Inc, Cary, NC, USA. SAS (r) Proprietary Software 9.2 (TS2M3)). 

## 3. Results

### 3.1. Preoperative and Intraoperative Data

Between January 1, 2006 and December 31, 2009, a total of 147 patients (135 appendiceal cancer, 12 colorectal cancer) were treated. There were 68 men and 79 women. The mean age was 49.9 years (range, 23–64). Data on patient characteristics, quantitative prognosis indicators, peritonectomies, visceral resections, and intraoperative treatments are summarized in Table 1. In these 147 patients, 424 peritonectomy procedures were performed with a mean of 3 peritonectomies per patient (range 0 to 5). In 18 patients (12%), no peritonectomy procedures were performed and 32 patients (22%) had all 5 peritonectomy procedures. A total of 455 visceral resections were performed. The mean number per patient was 3.1 with a range of 0 to 7. Greater omentectomy was performed in 144 patients (98%) and 4 (3%) had a total or partial gastrectomy. In two patients (1%), there were no visceral resections. Thirty-four (23%) had 2 visceral resections and an additional 34 patients (23%) had 3 visceral resections. The total number of anastomoses in all patients was 112 with a mean of 0.76 per patient (range 0–4). The most common anastomosis was a colorectal anastomosis performed in 56 patients (38%). There was a total of 43 ostomies (29%) performed. Thirty (20%) were diverting ileostomies to protect a colorectal anastomosis and, 13 (9%) were permanent-end ileostomies following total abdominal colectomy.

In 39 patients (27%), no blood replacement in the operating room occurred. Only 5 patients had more than six units transfused. Sixty-seven patients (46%) received fresh frozen plasma and, 16 (11%) patients had more than four units. All 147 patients were treated with HIPEC and intravenous 5-FU in the operating room. In 65 patients (44%), EPIC was used in the postoperative period (Table 1).

### 3.2. Adverse Events

In these 147 patients, there was a single postoperative death (0.7%). This patient developed a profound neutropenia followed by systemic inflammatory response syndrome and multiorgan failure. The overall incidence of gastrointestinal adverse events was 17%. Thirty-five gastrointestinal events occurred in 25 patients. Nine patients had more than one gastrointestinal event. All the gastrointestinal events observed grade I through IV are summarized in Table 2.

The incidence of grade III and IV events was 8% with 15 events observed in 12 patients. There were 5 grade I gastrointestinal events (4 pancreatitis and 1 chyle leak) that occurred in 5 patients. There were 15 grade II gastrointestinal events (4 pancreatitis, 1 prolonged ileus, 7 nausea/vomiting, and 1 diarrhea) that occurred in 11 patients. Four grade III gastrointestinal events (4 nausea/vomiting) occurred in 4 patients. Eleven grade IV gastrointestinal events (3 anastomotic failures, 3 fistulas, 1 pancreatic fistula, 1 pancreatitis, 1 bile leak, 1 small bowel obstruction, and 1 nausea/vomiting) occurred in 8 patients and all but two required a return to the operating room.

By univariate analysis, the following variables were proven to have a statistically significant correlation with gastrointestinal morbidity (Table 3): histological grade (*P* = 0.0166), PCI (*P* = 0.0049), small bowel resection (*P* = 0.0493), performance of a colorectal anastomosis (*P* = 0.0430) and the number of anastomoses performed per patient (*P* = 0.0288). 

On multivariate analysis using the logistic regression model, the PCI was shown to be the only independent risk factor for gastrointestinal complications (*P* = 0.0586).

In patients who had HIPEC plus EPIC, an additional four treatments with intraperitoneal 5-fluorouracil were given on postoperative days 1–4. Thirteen of 52 patients (25%) had a grade I–IV complication. Ten of 69 patients (14%) who had HIPEC only were observed to have a grade I–IV complication. As shown in Table 3, this was not significant by a univariate (*P* = 0.2314) or by the multivariate analysis.

## 4. Discussion

At our institution the management of peritoneal surface malignancy requires an integration of extensive surgery combined with intraperitoneal chemotherapy administered as a planed part of the surgical procedures [31]. The aim of this combined treatment modality is to remove all macroscopic tumor nodules and any adhesions between the bowel loops, in order to allow chemotherapeutic agents to be uniformly distributed within the peritoneal cavity to eradicate any microscopic tumor deposits. The potential advantages of using HIPEC compared to standard intravenous chemotherapy include an increased exposure to chemotherapeutic drugs at the peritoneal surface, an increase of drug penetration into the tissues, a synergistic effect of hyperthermia with systemic chemotherapy, and an independent cytotoxic effect of hyperthermia [32].

It is clear, however, that the effects of this regional chemotherapy are not limited to the peritoneal space. The profound effect that these treatments have on wound healing is shown by the increased incidence of gastrointestinal events. This paper represents the effort of our group to identify gastrointestinal events in patients with peritoneal surface malignancy and begin to understand their causes.

We found that our overall incidence of gastrointestinal events was 17% (grade I–IV), in that 35 gastrointestinal events occurred in 25 of the 147 patients. There was often more than one gastrointestinal event per patient. The incidence of grade III and IV gastrointestinal events was 8%.

Our data is compared to those reported by other authors in Table 4. We have calculated the incidence of gastrointestinal events (grades III-IV) in Glehen, Kusamura, and Hansson's manuscripts by dividing the total number of events by the total number of patients. In Youssef manuscript and in our data, we were able to calculate the incidence of events per patient (Table 4). Glehen et al. conducted a study of 207 patients treated by CRS and HIPEC with the closed abdominal technique [23]. The overall postoperative morbidity rate including all grades III-IV was 24.5%. They had 14 digestive fistulas, 11 cases of prolonged ileus, and 5 intraperitoneal abscesses. The presence of digestive fistula was significantly associated with the duration of surgery and the number of anastomoses in the univariate analysis. Kusamura et al. conducted a study of 205 patients treated by CRS and HIPEC with the closed abdominal technique [24]. The overall postoperative morbidity rate including all grade III-IV was 12%. They had 17 anastomotic leaks, 6 digestive perforations, 1 biliary fistula, 2 pancreatic fistulas, and 4 ileus/gastric stasis. The most severe complications in their series were intestinal leakage due to anastomotic insufficiency and/or intestinal perforation. This morbidity constituted approximately 70% of all cases with major morbidity. The rate of fistula in the series was 11%. They found in the multivariate analysis that the extent of cytoreduction (levels 1 and 2 versus 3) and CDDP for IPHP dose ≥ 240 mg were independent risk factors for major morbidity. 

Hansson et al. conducted a study of 123 patients treated by CRS and HIPEC and observed grade III-IV adverse events in 51 patients (41%) [25]. In multivariate analyses, grade III-IV adverse events were associated with stoma formation, duration of surgery, perioperative blood loss, and peritoneal cancer index. Among the gastrointestinal events, 7 anastomotic leaks, 11 digestive tract perforations, 1 pancreatitis, 1 bile leak, and 3 prolonged ileus occurred.

Youssef et al. conducted a study of 456 patients with pseudomyxoma peritonei syndrome of appendiceal origin [26]; grade III-IV morbidity was 7%. Seven anastomotic leaks, 5 pancreatic complications, and 8 intestinal fistulas were reported. An analysis of prognosis risk factors was not provided.

In our study, we found that the histological grade, PCI, small bowel resection, colorectal anastomoses performed, and the number of anastomoses performed per patient had a statistically significant correlation with gastrointestinal morbidity; by multivariate analysis only, the PCI was an independent risk factor for gastrointestinal complications. These data suggest that CRS combined with a standardized treatment with perioperative chemotherapy is a reasonable safe treatment for selected patients with peritoneal surface malignancy. There is an acceptable gastrointestinal morbidity as compared with modern series of pancreatic-duodenectomy, gastrectomy for cancer, or other multiorgan resections.

## Figures and Tables

**Table 1:** Patients characteristics, quantitative prognosis indicators, peritonectomies, visceral resections, and intraoperative treatments.

Variable	*n*
Total Patients	147
Gender	
Male	68 (46%)
Female	79 (54%)
Age at the time of surgery (years)	
Mean ± 1 Standard Deviation	49.9 (±8.7)
Median	51
Range	23–64
Primary cancer diagnosis	
Appendiceal cancer	135 (92%)
Colorectal cancer	12 (8%)
Histological grade	
Grade 1	61 (41.6%)
Grade 2	12 (8%)
Grade 3	74 (50.4%)
Prior surgical score	
0–2	133 (90.5%)
3	14 (9.5%)
Peritoneal cancer index	
0–10	31 (21%)
11–20	40 (27%)
21–30	53 (36%)
31–39	23 (16%)
Completeness of cytoreduction	
Complete	125 (85%)
Incomplete	22 (15%)
Peritonectomy procedures	
Pelvic	123 (84%)
Right upper quadrant	109 (74%)
Left upper quadrant	72 (49%)
Omental bursa	70 (48%)
Anterior abdominal wall	50 (34%)
Peritonectomy procedures per patient	
zero	18 (12%)
one	21 (14%)
two	16 (11%)
three	29 (20%)
four	31 (21%)
five	32 (22%)
Visceral resections performed	
Greater omentectomy	144 (98%)
Splenectomy	84 (57%)
Rectosigmoid colon resection	70 (48%)
Right colon resection	57 (39%)
Hysterectomy	47 (32%)
Small bowel resection	29 (20%)
Transverse colon resection	20 (14%)
Gastrectomy	4 (3%)
Visceral resections per patient	
Zero	2 (1.4%)
One	20 (13.6%)
Two	34 (23.1%)
Three	34 (23.1%)
Four	32 (21.8%)
Five	15 (10.2%)
Six	8 (5.4%)
Seven	2 (1.4%)
Anastomoses performed	
Esophageal	2 (1.4%)
Small bowel	22 (15.0%)
Ileocolic	29 (19.7%)
Colocolic	3 (2.0%)
Colorectal	56 (38.1%)
Anastomoses performed per patient	
Zero	74 (50.3%)
One	44 (29.9%)
Two	21 (14.3%)
Three	6 (4.1%)
Four	2 (1.4%)
Ostomies performed	
Diverting ileostomy	30 (20.4%)
End ileostomy	13 (8.8%)
None	67 (70.8%)
Blood replacement	
None	39 (26.5%)
Blood 1–3	68 (46.3%)
Blood 4–6	35 (23.8%)
Blood > 6	5 (3.4%)
Fresh frozen plasma replacement	
None	80 (54.4%)
Plasma 1–4	51 (34.7%)
Plasma >4	16 (10.9%)
Time in operating room (hours)	
0–6	10 (6.89%)
6–12	124 (84.4%)
> 12	13 (8.8%)
Chemotherapy treatments	
HIPEC only	82 (55.8%)
HIPEC plus EPIC	65 (44.2%)

**Table 2:** The total number of gastrointestinal adverse events grade I through grade IV.

Organ System	Grade I-Asymptomatic and self-limiting	Grade II-Symptomatic and medical treatment	Grade III-Invasive intervention	Grade IV-ICU care or return to operating room
Gastrointestinal System	*N* = 5	*N* = 15	*N* = 4	*N* = 11
Anastomotic failure	Sub-clinical, afebrile, radiologic diagnosis (0)	Antibiotics, febrile (0)	Percutaneous drainage (0)	Reoperation (3)
Fistula	Sub-clinical, afebrile, radiologic diagnosis (0)	Antibiotics, febrile (0)	Percutaneous drainage (0)	Reoperation (3)
Pancreatic fistula	Elevated enzymes in drains (0)	TPN and somatostatin (0)	Percutaneous drainage (0)	Reoperation (1)
Pancreatitis	Elevated enzymes (4)	≤3 Ranson's score (4)	4–6 Ranson's score (0)	Reoperation/ICU (1)
Bile leak	Bile only in drain (0)	Bile in drain, febrile (2)	Percutaneous drainage (0)	Reoperation (1)
Chyle leak	Transient (1)	Prolonged 1 week (0)	Cease prior to discharge (0)	Persist past hospital discharge (0)
Prolonged ileus	N/G (0)	N/G > 2 weeks (1)	N/G >3 weeks (0)	Persist past hospital discharge (0)
Small bowel obstruction	Abdominal pain (0)	Abdominal pain, N/G reinsertion (0)	Repeat radiologic studies (0)	Reoperation (1)
Nausea/vomiting	Transient vomiting (0)	Vomiting, anti-emetics (7)	Vomiting, IV therapy (4)	Vomiting with surgical intervention or ICU (1)
Diarrhea	Transient <2 days (0)	Tolerable, but >2 days (1)	Intolerable, IV therapy (0)	Dehydration prolonged IV therapy (0)
Ascites	Mild (0)	Fluid restriction (0)	Symptomatic, percutaneous tap (0)	Compromising vital function, ICU care (0)

**Table 3:** Univariate and multivariate analysis (gastrointestinal events).

	Gastrointestinal events I–IV univariate analysis			Gastrointestinal events I–IV multivariate analysis	
	Yes *N* = 25	No *N* = 122	*P* Value*/OR (95% CI)	Odds Ratio	*P* Value
Gender					
Male	12	56	0.8480		NT**
Female	13	66	0.9 (0.4,2.2)		
Age					
≤50 year	14	58	0.4408		NT
>50 year	11	64	0.7 (0.3,1.7)		
Location					
Appendix	22	113	0.4297		NT
Colorectal	3	9	1.7 (0.4,6.8)		
Grade					
Grade 1	5	56	0.0166	0.4(0.1, 1.3)	0.1345
Grades 2-3	20	66	0.3 (0.1,0.8)		
Prior Surgical Score					
0–2	23	110	1.0000		NT
3–5	2	12	0.8 (0.2,3.8)		
Peritoneal Cancer Index (4 groups)					
0–10	3	28	Reference		NT
11–20	6	34	0.7220 1.6 (0.4,7.2)		
21–30	7	46	0.7384 1.4 (0.3,5.9)		
31–39	9	14	0.0100 6.0 (1.4,25.7)		
Peritoneal Cancer Index (2 groups B)					
0–30	16	108	0.0049	2.8 (0.9,8.4)	0.0586
31+	9	14	4.3 (1.6,11.7)		
Completeness of Cytoreduction					
Complete	18	107	0.0625		NT
Incomplete	7	15	2.8 (1.0,7.7)		
Peritonectomy Procedure					
Pelvic	21	102	1.0000 1.0 (0.3,3.3)		NT
Right Upper Quardrant	19	90	0.8166 1.1 (0.4,3.1)		NT
Left Upper Quardrant	11	61	0.5846 0.8 (0.3,1.9)		NT
Omental Bursa	12	58	0.9666 1.0 (0.4,2.4)		NT
Anterior Abdominal Wall	11	39	0.2473 1.7 (0.7,4.0)		NT
Peritonectomy Procedure per patient					
0–2	8	47	0.5391		NT
3–5	17	75	1.3 (0.5,3.3)		
Visceral Resections Performed					
Omentectomy	24	120	0.4308 0.4 (0.03,4.6)		NT
Splenectomy	15	69	0.7513 1.2 (0.5,2.8)		NT
Rectosigmoid colon	14	43	0.0524 2.3 (0.98,5.6)		NT
Right colon resection	15	55	0.1736 1.8 (0.8,4.4)		NT
Hysterectomy	4	43	0.0601 0.3 (0.1,1.1)		NT
Small bowel resection	9	20	0.0493 2.9 (1.1,7.4)	1.3 (0.4,4.2)	0.6202
Transverse colon	5	15	0.3377 1.8 (0.6,5.5)		NT
Gastrectomy	2	2	0.1343 5.2 (0.7,38.9)		NT
Visceral Resections Performed per patient					
0–2	7	49	0.2539		NT
3–7	18	73	1.7 (0.7,4.4)		
Anastomoses performed					
Esophago-jejunal	1	1	0.3122 5.0 (0.3,83.4)		NT
Small bowel	7	15	0.0605 2.8 (1.0,7.7)		NT
Ileocolic	7	22	0.2741 1.8 (0.7,4.7)		NT
Colocolic	1	2	0.4308 2.5 (0.2,28.7)		NT
Colorectal	14	42	0.0430 2.4 (1.0,5.8)	1.4 (0.5,3.8)	0.4986
Anastomoses performed per patient					
0–2	21	118	0.0288	1.9 (0.3,11.8)	0.4616
3–5	4	4	5.6 (1.3,24.2)		
Ostomies performed					
None	15	89	reference		
Diverting ileostomy	8	22	0.1172 2.2 (0.8,5.7)		NT
End ileostomy	2	11	1.0000 1.1 (0.2,5.4)		NT
Blood replacement					
None	7	32	reference		
Blood 1–3	8	60	0.3752 0.6 (0.2,1.8)		NT
Blood 4–6	9	26	0.4178 1.6 (0.5,4.8)		NT
Blood > 6	1	4	1.0000 1.1 (0.1,11.8)		NT
Fresh frozen plasma replacement					
None	13	67	reference		
Plasma 1–4	7	44	0.6953 0.8 (0.3,2.2)		NT
Plasma > 4	5	11	0.1724 2.3 (0.7,7.9)		NT
Time in operating room (hours)					
0–6	2	8	reference		
7–12	18	106	0.6445 0.7 (0.1,3.5)		NT
>12	5	8	0.450 2.3 (0.4,16.9)		NT
Chemotherapy treatment					
HIPEC only	10	69	0.2314		NT
HIPEC plus EPIC	13	52	1.7 (0.7,4.2)		
Unknown	2	1			

*: Pearson Chi-square, or Fisher's exact test if sparse distribution. **: NT means Not Tested in multivariate modeling due to non significant univariate test.

**Table 4:** Comparison with other series with more than 100 patients.

Author	Institution	Year	Patient revised	Histology	GI Events Revised	GI Morbidity Grades III-IV	Risk Factor for GI Events	Statistical Analysis
Glehen et al. [23]	Lyon	2003	207	Colon, PMP, ovarian	Digestive fistula (14) Prolonged ileus (11) Intraperitoneal abscess (5)	*15% 30 events	Carcinomatosis stage Duration of surgery Number of anastomosis	Univariate
Kusamura et al. [24]	Milan	2006	205	PM, PMP, ovarian	Anastomotic leak (17) Digestive perforations (6) Biliary fistula (1) pancreatic fistula (2) Ileus/gastric stasis (4)	*15% 30 events	Extent of cytoreduction CDDP IPHP dose	Multivariate
Hansson et al. [25]	Uppsala	2009	123	Colorectal, PMP, PM, ovarian	Anastomotic leak (7) Digestive perforation (11) Pancreatitis (1) Bile leak (1) Ileus (3)	*19% 23 events	Stoma formation Duration of surgery Perioperative blood loss PCI	Multivariate
Youssef et al. [26]	Basingstoke	NP	456 Just 441 had resections	PMP (appendix)	Anastomotic leak (7) Pancreatic complicate (5) Intestinal fistula (8)	4.5% 20 events	NA	
Present series	Washington	2011	147	Colon, PMP (appendix)	Anastomotic failure (2) Fistula (4) Pancreatic fistula (1) Pancreatitis (1) Bile leak (1) Chyle leak (0) Prolonged ileus (0) Small bowel obst (1) Vomiting (5) Diarrhea (0) Ascites (0)	8% 15 events	Grade PCI Small bowel resection Colorectal anastomoses Number of anastomosis per patient	Multivariate

* Percentage calculated from total number of events/total number of patients.

NP: Not published. NA: Not available. CDDP: cisplatin, IPHP: intraperitoneal hyperthermic perfusion. PM: peritoneal mesothelioma. PMP: pseudomyxoma peritonei
